# Toward Corneal Limbus In Vitro Model: Regulation of hPSC‐LSC Phenotype by Matrix Stiffness and Topography During Cell Differentiation Process

**DOI:** 10.1002/adhm.202301396

**Published:** 2023-07-21

**Authors:** Maija Kauppila, Anni Mörö, Juan José Valle‐Delgado, Teemu Ihalainen, Lassi Sukki, Paula Puistola, Pasi Kallio, Tanja Ilmarinen, Monika Österberg, Heli Skottman

**Affiliations:** ^1^ Faculty of Medicine and Health Technology Tampere University Tampere 33520 Finland; ^2^ Department of Bioproducts and Biosystems School of Chemical Engineering Aalto University Espoo 02150 Finland

**Keywords:** corneas, human pluripotent stem cells, in vitro investigations, limbus, mechanobiology

## Abstract

A functional limbal epithelial stem cells (LSC) niche is a vital element in the regular renewal of the corneal epithelium by LSCs and maintenance of good vision. However, little is known about its unique structure and mechanical properties on LSC regulation, creating a significant gap in development of LSC‐based therapies. Herein, the effect of mechanical and architectural elements of the niche on human pluripotent derived LSCs (hPSC‐LSC) phenotype and growth is investigated in vitro. Specifically, three formulations of polyacrylamide gels with different controlled stiffnesses are used for culture and characterization of hPSC‐LSCs from different stages of differentiation. In addition, limbal mimicking topography in polydimethylsiloxane is utilized for culturing hPSC‐LSCs at early time point of differentiation. For comparison, the expression of selected key proteins of the corneal cells is analyzed in their native environment through whole mount staining of human donor corneas. The results suggest that mechanical response and substrate preference of the cells is highly dependent on their developmental stage. In addition, data indicate that cells may carry possible mechanical memory from previous culture matrix, both highlighting the importance of mechanical design of a functional in vitro limbus model.

## Graphical abstract

For the development of native tissue mimicking limbus in vitro model, human pluripotent stem cell derived limbal epithelial cells are cultured on varying stiffness polyacrylamide gels and polydimethylsiloxane matrix with limbus mimicking topography, demonstrating that substrate preference is highly dependent on their developmental stage. The results highlight the importance of mechanical design of a culture substrate.

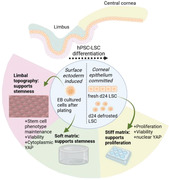

## 1 Introduction

Functional corneal limbal epithelial stem cells (LSCs) are of vital importance for maintenance of good vision. LSCs are responsible for the regular renewal of the cornea epithelium and in native human cornea, these cells reside in limbus which is a distinct anatomical location in the peripheral cornea. In limbus, an individual pattern of radially oriented interpalisade ridges called Palisades of Vogt, extend into limbal epithelial crypts in which LSCs reside.^[^ ^1^ ^]^ These crypts, together with a complex set of chemical and biological factors, such as blood vessels and other niche cells, provide a unique microenvironment (niche) for LSCs. The microenvironment maintains the stemness of LSCs as well as provides signals and cues related to proliferation, migration and differentiation toward corneal epithelial cells and fully matured stratified corneal epithelium.^[^ ^2^ ^]^ Destruction of limbus leads to dysfunction of LSCs and cease of epithelium renewal, highlighting the indispensable role of the physicochemical niche in LSC regulation.^[^ ^3^ ^]^ Thus, modeling the niche environment in vitro would give valuable insights for cellular therapies and LSC related disease pathologies including aniridia.^[^ ^4^ ^]^

Despite the identification of LSC's native niche, little is known about its regulatory effect on LSC maintenance, corneal homeostasis, and spatial organization of the cells in vivo. A major drawback in LSC research is a lack of a specific biomarker on identification of true LSCs among variety of cell types present in limbus. However, recent studies with high‐throughput RNA sequencing techniques have unraveled new cellular markers for LSCs.^[^ ^5^, ^6^, ^7^, ^8^ ^]^ Importantly also, these studies have emphasized that in native niche, the stem cell pool is composed of several heterogenous LSC subpopulations with possibly different regeneration capacities.^[^ ^8^ ^]^ To study these subpopulations, human pluripotent stem cells (hPSC) offer a unique development biology mimicking tool to produce various types of cells via controlled differentiation from undifferentiated hPSC toward mature cornea epithelium. In our previous studies, we have demonstrated the capability to produce high‐quality hPSC‐LSCs in xeno‐free conditions.^[^ ^9^, ^10^ ^]^

However, currently used standard two dimensional (2D) in vitro culture substrates with unphysiological stiffness are not sufficient to mimic native limbal environment and pass over the important regulatory effect of the physical niche. Recent studies have highlighted the importance of the surrounding mechanical environment in LSC regulation, mediated by the YES‐associated protein (YAP) signaling both in human and murine corneas.^[^ ^11^, ^12^, ^13^, ^14^, ^15^ ^]^ It has been shown that the middle and basal layer of the limbal epithelium provide significantly softer environment for LSCs than the central cornea,^[^ ^12^ ^]^ and accordingly, previous studies have shown capability to modulate primary LSC phenotype through matrix stiffness in vitro.^[^ ^13^ ^]^ However, it is not clear how distinct mechanical environment affects subpopulations of LSCs at different stages of cellular differentiation or at different location in the limbal‐corneal path. Moreover, previous studies show that surface topography, another important feature in limbal niche, has potential to regulate the behavior of corneal cells.^[^ ^16^ ^]^ Previous mechanobiological studies have utilized isolated human and mouse primary cell cultures or immortalized cell lines and to our knowledge, no studies have been conducted with hPSC‐LSCs with possibility to study mechanobiology with different developmental states of LSCs.^[^ ^11^, ^12^, ^44^ ^]^

Polyacrylamide gels (PA) are widely used platforms to study mechanobiology since they offer multiple advantages in cellular studies including translucency and nearly linear elastic behavior. In addition, there are well‐established protocols to produce these gels with a wide range of stiffnesses, including low moduli mimicking soft tissue stiffnesses (>10 kPa).^[^ ^17^ ^]^ As an alternative to traditional coating methods, a 4‐dihydroxy‐l‐phenylalanine (L‐DOPA) based coating provide an excellent alternative for surface functionalization with a capability to create a stiffness independent, uniform coating on varying stiffness PA gels.^[^ ^18^ ^]^ In addition, polydimethylsiloxane (PDMS) has been widely explored in cellular studies and exhibits several advantages, such as easy formability.^[^ ^19^ ^]^ In here, we utilized PA gels and PDMS to study two important features of the physical limbal niche, stiffness, and topography, with varying stiffness PA gels and PDMS platform with limbus‐mimicking topography.

**Figure 1:**
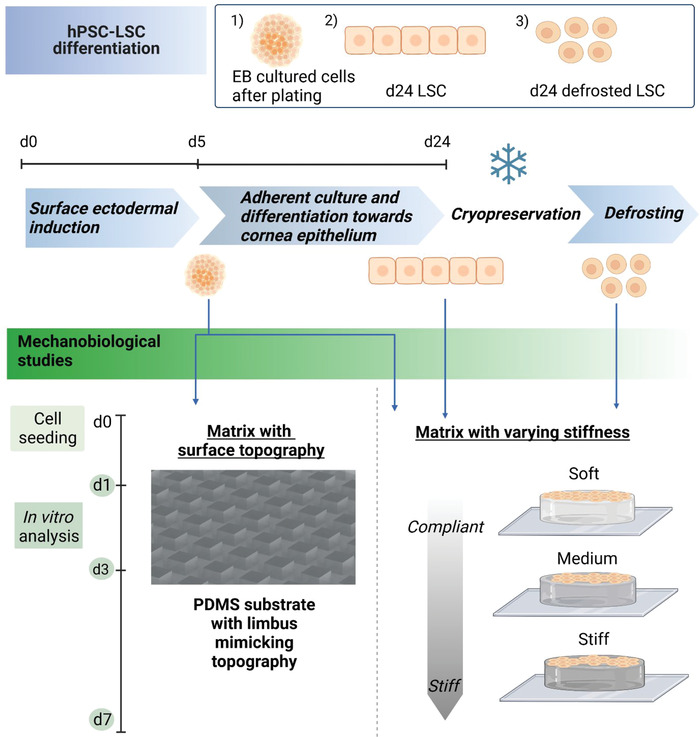
Schematic representation of the study. Image created with Biorender.com.

In this study, we aimed to investigate how matrix stiffness, extracellular matrix (ECM) components and topography affect hPSC‐LSCs growth and phenotype during their differentiation process mimicking limbal niche conditions. We selected three cell populations from hPSC‐LSCs differentiation trajectory to investigate how the developmental stage of LSCs affect their mechanobiological responses. Specifically, these compared cell populations included (see **Figure** **1**); 1) Day 5 suspension cultured cells in embryoid bodies (EB) after plating (early time point from differentiation, subjected to surface ectodermal induction); 2) Day 24 hPSC‐LSCs (late time point from differentiation, cornea epithelium committed) as well as 3) the cryopreserved and defrosted d24 hPSC‐LSC population. We successfully cultured these three developmentally different hPSC‐LSCs subpopulations on varying stiffness L‐DOPA+ECM protein functionalized PA gels and limbus‐topography mimicking PDMS structures and compared their relevant marker expression to native human limbal cells in vivo by whole mount staining's.

## 2 Results and Discussion

### 2.1 In Vivo Validation of Key Corneal Cell Markers

**Figure 2:**
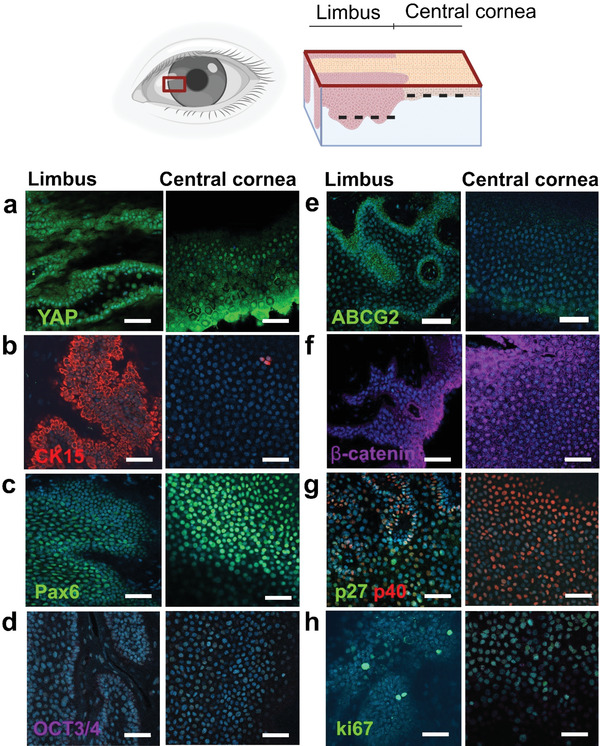
Whole mount immunofluorescence staining of human donor corneas imaged with a confocal microscope for validation of key marker expression in vivo. Illustration of cut tissue sample from human donor cornea indicates whether image is taken from limbal area (limbus) or near basal layer of central cornea (illustration created with Biorender.com). Analyzed markers are a) YAP, b) CK15, c) Pax6, d) OCT3/4 (no expression), e) ABCG2, f) Beta‐catenin, g) p27 and p40 and h) ki67. Nuclei were counterstained with Hoechst. Scalebars are 50 µm.

To understand the effect of the matrix stiffness and topography on hPSC‐LSCs, a key question is how the hPSC‐LSCs resemble LSCs in their native environment and for comparison, how the different mechanical properties from limbus to central cornea effect LSC phenotype in vivo. Mechanical properties of the cornea have been reported with different analysis methods, such as Brillouin spectroscopy and atomic force microscope (AFM) measurements by several groups, all in agreement with the gradual stiffening of the cornea from its limbal edges to the center.^[^ ^13^, ^20^, ^21^ ^]^ Despite of distinct anatomical compartments segregating LSCs and differentiated cells and creation of a single cell RNA sequencing atlas of human corneal cells,^[^ ^8^ ^]^ a clear view of corneal hierarchy has not been established. As accurate localization of the cells in their native environment is essential for comparison with cells cultured in vitro, efficient high spatial resolution analysis methods of the native cornea is needed. Although cornea and limbus are easily accessible, limitations in histological research of human corneas arise from shortage and heterogeneity of the human donor tissue. In addition, heterogeneity of the biomarkers and their combinations used in identification of LSCs and progenitor cells has set a challenge to obtain a comprehensive view on the corneal hierarchy.^[^ ^22^ ^]^ Thus, we initiated our studies by determination of key corneal marker expression with the same antibodies that were used for in vitro characterization of hPSC‐LSC. We performed whole mount immunofluorescence staining's for human donor corneas with optical tissue clearing and compared marker expression in limbal crypts as compared to basal layer of central cornea (**Figure** **2**; and Figures S1–S8, Supporting Information).

Whole mount immunofluorescence is widely utilized for murine corneas with well‐established protocols,^[^ ^23^ ^]^ but for human cornea only few reports were found.^[^ ^24^, ^25^ ^]^ Thin tissue sections are used as a golden standard for histological analysis of the human cornea, but they lack the spatial information from the sample which is paramount in identification of different LSC populations in their native environment. Identification of different cells throughout the cornea from thin sections is demanding and laborious.^[^ ^26^ ^]^ Thus, whole mount immunofluorescence is a superior method compared to traditional immunofluorescence from histological sections since it preserves intact tissue architecture and cellular organization and provides a possibility to analyze the marker expression from a large piece of a cornea at once.

Whole mount staining's confirmed that in soft limbus, cells were expressing well‐acknowledged LSC markers cytokeratin 15 (CK15), ATP binding casette subfamily G member 2 (ABCG2) and p40, a ΔNp63 isoform (Figure 2b,e,g),^[^ ^27^ ^]^ and these markers were showing negative or only low expression in central cornea except for p40. However, ΔNp63α is the only truly specific isoform for limbal cells in the human cornea and γ and β isoforms are present also in the central cornea.^[^ ^28^ ^]^ This suggests that cells expressing p40 in the central cornea are representing these two latter isoforms since a specific isoform was not verified due to the lack of a commercially available antibody. This is also supported by absence of CK15 and ABCG2 in the central cornea. Whole mount staining also confirmed absence of pluripotent marker octamer‐binding transcription factor (OCT) 3/4 and positive expression of paired box protein 6 (PAX6), a key marker in eye development, throughout the cornea (Figure 2c,d). Although previous studies have showed Oct4‐positive limbal niche cells,^[^ ^29^ ^]^ we could not see those cells in our sample at least with our antibody. Interestingly, in line with a work by Gouveia et al.,^[^ ^13^ ^]^ YAP remains mainly cytoplasmic in softer limbus and translocate in the cell nucleus in stiffer central cornea (Figure 2a; and Figure S1, Supporting Information). In addition, YAP is located in cellular junctions in the central cornea. Expression pattern of ki67 and p27, indicating cellular proliferation and quiescence respectively, show exclusive expression of p27 in limbus (Figure 2g) and interestingly, ki67 shows most prominent expression in the basal layer of the limbal/corneal transition zone (Figure 2h; and Figure S4, Supporting Information). The results indicate cell population with highest proliferation extending from the limbal crypts to central cornea which is further supported by nuclear expression of β‐catenin, a pro‐proliferative marker, in some of the basal cells of the central cornea (Figure 2f). Overall, the whole mount staining results also support a hypothetical hPSC‐LSC differentiation hierarchy previously published by our group, where quiescent (ABCG2/p27 positive) hPSC‐LSCs turn first into actively proliferating ΔNp63α and CK15 positive cells before finally adapting a cytokeratin 12 (CK12) positive mature corneal cell phenotype.^[^ ^30^ ^]^ Importantly, we obtained a relevant comparison of LSCs in their native environment for the following experiments.

### 2.2 Determination of Bulk and Local Stiffness of Varying Stiffness PA Gels

**Figure 3:**
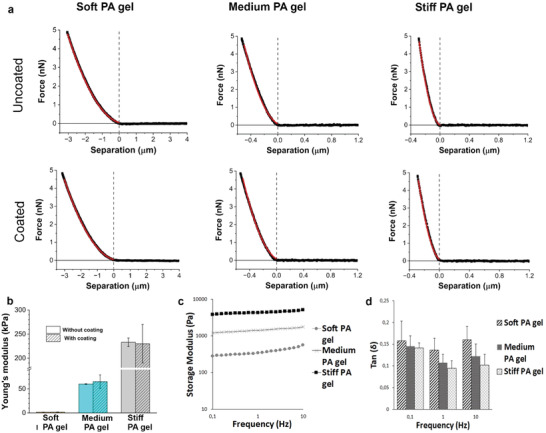
Mechanical characterization of PA‐gels with atomic force microscopy (AFM) and oscillatory rheology. a) Representative AFM indentation curves for PA gels without (top row) and with (bottom row) protein coating. The indentation of the samples is shown as negative separation distances. Red lines correspond to theoretical fits according to the Sneddon model. b) Comparison of the Young's modulus obtained with AFM of different PA gels with and without protein coating, confirming that coating does not affect local stiffness significantly. Mean values and standard deviations (error bars) are shown (30 ≤ *n* ≤ 60). c) Storage modulus (*G*’) of PA gels obtained from oscillatory frequency sweep at 0.1–10 Hz are in good agreement with AFM measurements. d) Tan(*δ*) values for PA gels at 0.1, 1, and 10 Hz with standard deviations, showing nearly ideally elastic behavior of the PA gels.

The 3D geometry of a native tissue is an important regulator of cellular behavior, which is not met in standard cell culture on a plastic dish.^[^ ^31^ ^]^ The native tissue and especially stem cell niches exhibit complex mechanical behavior and simplified models are often utilized in in vitro studies.^[^ ^32^ ^]^ To evaluate how varying matrix stiffness alone affects hPSC‐LSC growth and phenotype in a 2D‐culture, we prepared PA gel matrix with three stiffness’ (soft, medium, and stiff PA gel) and compared them with a glass matrix. PA gel surface was functionalized with L‐DOPA and ECM protein coating that is used in our standard hPSC‐LSC differentiation on tissue culture plastic.^[^ ^10^ ^]^ To control if functionalization with large ECM protein caused increase in local stiffness and mechanical properties of the PA matrices used, we validated the local mechanical properties of the three stiffness matrices with and without surface functionalization and mechanical properties of the whole bulk material. For this, atomic force microscopy (AFM) and oscillatory rheology measurements were used for determination of local and global stiffness, respectively (**Figure** **3**).

We characterized Young's modulus (*E*) of different stiffness PA‐gels and obtained *E* = 1.6 ± 0.3 kPa without coating and *E* = 1.7 ± 0.4 kPa with coating for soft PA gel, *E* = 60 ± 1 kPa without coating and *E* = 65 ± 14 kPa with coating for medium PA gel and *E* = 233 ± 9 kPa without coating and *E* = 230 ± 40 kPa with coating for stiff PA gel (Figure 3b). Most importantly, AFM measurements show that L‐DOPA+ECM protein conjugation on PA gel surface has no significant effect on local stiffness values compared to uncoated gels. In comparison with a native cornea, Kazaili et al. reported Young's modulus *E* = 228 ± 11 kPa for a porcine cornea at a normal intraocular pressure (IOP, 15 mmHg) with a decrease by 11.5% in the limbal region with oscillatory nanoindentation, corresponding to the stiffest PA gel.^[^ ^21^ ^]^ Last et al. have reported *E* = 7.5 ± 4.2 kPa for anterior basement membrane by AFM measurements, closest to the softest PA gel.^[^ ^20^ ^]^ Previous studies have also showed increase of Young's modulus within aging from human donor corneas: Knox Cartwright et al. showed increase of Young's modulus by a factor of approximately two between the ages of 20 and 100 years, and this tissue stiffening has also been associated with decreasing capability of corneal regeneration.^[^ ^33^ ^]^ In here, AFM verified that we could produce PA gels with physiologically relevant stiffness scale.

PA‐gel surface itself has no cell‐binding motifs or binding sites for proteins and traditionally, gel surface is functionalized with a chemical crosslinker sulfosuccinimidyl 6‐(4′‐azido‐2′‐nitrophenylamino)hexanoate (sulfo‐SANPAH). However, sulfo‐SANPAH exhibits several disadvantages in surface functionalization, such as poor solubility and limited stability.^[^ ^34^ ^]^ These may lead variations in cross‐linking and heterogeneity of the ECM coating and thus, varying number of binding sites for cells. Consequently, this may lead to misinterpretations on cellular response to matrix stiffness. In here, PA gels with L‐DOPA+ECM coating is proven to be an excellent option for mechanobiological studies since this surface functionalization method provides a uniform coating without affecting stiffness values of the substrate.

After characterization of local stiffness values, we analyzed global mechanical properties of the uncoated PA gel in different stiffness’ with shear rheology (Figure 3c,d). We utilized oscillatory rheology with small amplitude shear oscillation which is a widely used method to give insight on viscoelastic properties of hydrogels among many other relevant properties such as gelation kinetics.^[^ ^35^ ^]^ Frequency sweep can be utilized to segregate elastic portion (storage modulus *G’*) and viscous portion (loss modulus, *G’’*) of the viscoelastic behavior over a frequency range.^[^ ^36^ ^]^ At 0.1 Hz, storage modulus (*G’*) values for PA gels from soft to stiffest are 281, 1196, and 3818 Pa, respectively. The results correlate with the AFM measurements, confirming the stiffness range spaced at approximately similar intervals and we verified homogenous crosslinking of the gels, shown as consistent *G’* values over the frequency range.

PA gels have been previously shown to exhibit nearly ideal elastic behavior with a linear relationship of stress and strain, which is manifested by significantly higher *G’* values than *G’’* values.^[^ ^37^ ^]^ However, as a hydrogel PA gel exhibits also viscous behavior deriving from the inner water flow and thus, poroelastic model have been suggested to be applied to describe PA gel mechanical behavior.^[^ ^38^ ^]^ Soft biological tissues exhibit both viscoelastic and poroelastic components in their response to a load, deriving from rearrangement of the ECM and corresponding resistance of a fluid flow in a porous ECM.^[^ ^39^ ^]^ This complex behavior, mainly attributed by its viscoelastic component, has been recently shown to influence on several important cellular processes such as cell spreading and stress fiber formation.^[^ ^40^ ^]^ This is also true for the cornea in which arrangement of collagen fibrils orchestrate the anisotropic viscoelastic behavior.^[^ ^41^ ^]^ In addition to frequency sweep, we plotted tan(*δ*), a ratio between storage and loss modulus, to evaluate viscoelastic behavior of the PA gels more in detail (Figure 3D) and we obtained tan (*δ*) = 0.16 ± 0.04 for soft PA gel, tan (*δ*) = 0.158 ± 0.02 for medium gel and tan (*δ*) = 0.14 ± 0.01 for stiff gel at 0.1 Hz, confirming dominating elastic properties of the PA gels. For porcine cornea under normal IOP, tan(*δ*) *=* 0.15 measured with oscillatory nanoindentation has been reported by Kazaili et al. which is of same magnitude as our results.^[^ ^21^ ^]^

It has been previously reported that despite of wide use in biomedical applications, mechanical characterization of PA gels is highly variable and dependent on the selected measuring technique, variables in gel synthesis and storage time.^[^ ^42^ ^]^ Indeed, highlighted by Megone et al., systematic comparison of gels prepared in identical conditions and with similar mechanical test setting is largely lacking.^[^ ^43^ ^]^ Thus, mechanobiological platforms should always be carefully validated. In here, we validated mechanical properties of the PA gels with two independent methods and demonstrated production of mechanically homogenous PA gels with varying stiffness independent on protein coating and similar stiffness values as the native cornea. Next, we used this validated model with the hPSC‐LSCs.

### 2.3 hPSC‐LSC Response to Substrate is Highly Dependent on their Developmental Stage

**Figure 4:**
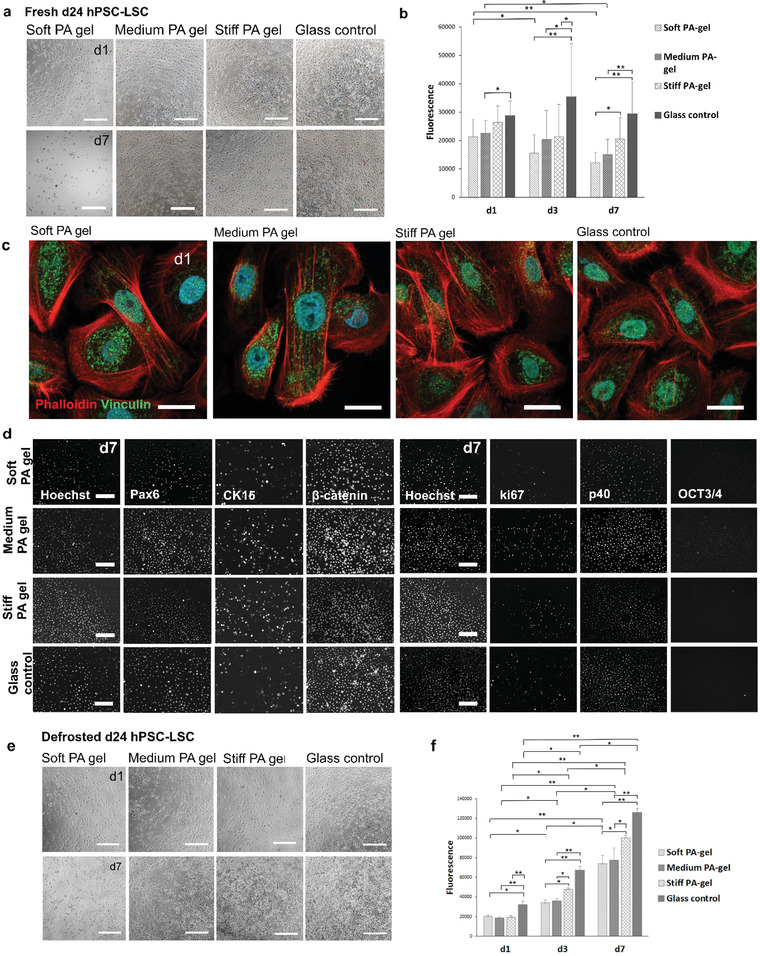
Characterization of d24 hPSC‐LSC growth and viability on different stiffness PA gels and glass control. a) Phase contrast microscopy images on fresh d24 hPSC‐LSC morphology on different stiffness’ at d1 and d7 demonstrate successful cell adherence. b) PrestoBlue viability assay results of fresh d24 hPSC‐LSC on different stiffness PA‐gels from d1 to d7 show highest viability on glass control. Statistical analysis was conducted with nonparametric Kruskal–Wallis H‐test (*n* = 7, ∗∗ = *p* < 0.001, * = *p* < 0.05). c) Immunofluorescence staining of actin binding protein vinculin (green) and actin cytoskeleton binding phalloidin (red) at d1 confirm formation of focal adhesions. d) Characterization of relevant LSC marker expression for fresh d24 hPSC‐LSCs. On left: PAX6, CK15, and β‐catenin, cell nuclei visualized with Hoechst. On right: of ki67, p40, OCT3/4. e) Morphology images from defrosted d24 hPSC‐LSC on different stiffness at d1 and d7. f) PrestoBlue viability assay results of defrosted d24 hPSC‐LSC on different stiffness PA gels and glass control from d1 to d7 show highest viability on the stiffest matrix consistently. Statistical analysis was conducted with nonparametric Kruskal–Wallis H‐test (*n* = 3, ∗∗ = *p* < 0.001, * = *p* < 0.05). Scalebars for images (a) and (d) are 200 µm and for image (c) 50 µm.

As previous studies have demonstrated that matrix stiffness is an important regulator of primary LSC phenotype in vitro and in vivo,^[^ ^11^, ^13^, ^44^ ^]^ regional mechanical environments may be critical in regulation of distinct subpopulations of LSCs which emphasizes the need to study the effect of different mechanical environments on different LSC subpopulations in vitro. In vitro differentiation of hPSC‐LSCs provides a unique opportunity to explore the effect of matrix stiffness on different time points on the controlled developmental trajectory. First, we utilized d24 hPSC‐LSCs, at a time point where our controlled in vitro differentiation reaches enriched population of p40 (∆Np63) positive LSC cells and cells are ready for cryostorage.^[^ ^10^ ^]^ In addition, we utilized d24 cells after cryostorage to see if cryopreservation alters the mechanobiological behavior of the cells. Freezing of the cells in general is a well‐established method for long‐term preservation however, it is known to induce cell stress.^[^ ^45^ ^]^ To the best of our knowledge, the effect of cryopreservation on cell mechanobiology has not been previously evaluated, at least not with LSCs. In here, we plated cells freshly from the adherent culture (fresh d24 hPSC‐LSC) or alternatively thawed cells from cryostorage (defrosted d24 hPSC‐LSC), directly on ECM functionalized PA gels with varying stiffness (soft, medium, and stiff) as well as on rigid glass control, and cultured cells for 7 days. During the culture we monitored carefully the hPSC‐LSC morphology, viability via metabolic activity, and adherence on the different matrices. To study phenotype and characteristics of the cells in detail, we performed extensive immunofluorescence staining's (**Figure** **4**).

Throughout spreading of fresh d24 hPSC‐LSC on PA gel surfaces indicate successful cell adherence and uniform distribution of protein coatings on the PA gel and glass surface (Figure 4a). PrestoBlue viability assay for fresh d24 hPSC‐LSCs showed no significant differences in viability on varying stiffness PA gels at d1 or d3. However, at d7 cell viability was significantly higher (*p* < 0.05) on stiff PA gel and glass control than on soft PA gel and hPSC‐LSCs on glass control also exhibited significantly higher (*p* < 0.001) viability than on medium PA gel (Figure 4b). The viability on glass drops from d3 to d7, which can be explained by confluency and decreased cell metabolic activity. Overall, the glass matrix supported d24 hPSC‐LSC viability best based on the PrestoBlue data.

Vinculin is a critical mechanosensitive adaptor protein, which mediate the integrin‐actin linkage together with talin, another adaptor protein.^[^ ^46^ ^]^ We observed vinculin expression pattern of d24 hPSC‐LSC seeding on different stiffness PA gels (Figure 4c) and localization of vinculin indicates the formation of functional focal adhesions on all matrices. However, no differences in the amount or localization of focal adhesions were observed between different stiffnesses. In glass control, cells exhibit more of triangular cell shape and less pronounced actin fibers as in all PA gels (Figure 4c).

Importantly also, we analyzed the effect of stiffness for the phenotype characteristics of the d24 hPSC‐LSCs. Based on these results, the marker expression with in vitro cultured cells verified that cells were expressing PAX6, p40, and CK15 without differences between conditions (Figure 4d). In addition, similar cell proliferation was verified with positive ki67 and β‐catenin between all conditions in addition to the downregulation of pluripotency with negative OCT3/4. Overall, no significant differences in marker expression were observed between the different matrices (Figure 4d). Compared to whole mount data (Figure 2), protein expression of d24 hPSC‐LSC is similar as in limbus observed ex vivo except for mainly nuclear expression of β‐catenin (with a few cells showing cytoplasmic expression on glass control) and high ki67 expression in PA gels, which resemble more the marker expression in limbal/corneal transition zone (Figure 2f,h; and Figure S4, Supporting Information). It has been previously shown that in primary LSCs, nuclear beta‐catenin is linked to high cellular proliferation in vitro, as we also could verify with high ki67 expression.^[^ ^47^ ^]^ Hence, d24 hPSC‐LSCs could potentially resemble active LSCs in inner limbus, a model of LSC populations in vivo described by Altshuler et al. in mouse.^[^ ^7^ ^]^ However, cell seeding and generation of a uniform cell layer may partly explain the phenomena, compared to a normal homeostatic condition in the donor cornea.

Interestingly, as we conducted same experiments for defrosted d24 hPSC‐LSCs, we noticed that results are not completely similar as compared to fresh d24 hPSC‐LSC. Defrosted hPSC‐LSCs attached readily on all stiffnesses, even on the softest PA‐gels (Figure 4e). However, similar trend as with fresh d24 cells could be observed from PrestoBlue data, showing significantly higher viability (*p* < 0.05) on glass and also on stiff PA gel compared to soft and medium PA gel at d7 (Figure 4f). In addition, viability increases significantly (*p* < 0.001) from d1 to d7 on all matrices (Figure 4f).

Interestingly, the results indicate that the d24 hPSC‐LSCs, both fresh and defrosted, which are differentiated on adherent culture on plastic, show highest viability on glass substrate despite of highly unphysiological matrix stiffness. In contrast, it has been reported by Masterton et al. that with immortalized human LSCs, viability was lower in plastic (stiff) than in PDMS with a stiffness range of 10–1500 kPa.^[^ ^11^ ^]^ We hypothesize that higher viability on rigid surfaces may be due to the priming of hPSC‐LSCs on a stiff matrix during their 2D differentiation as it has been shown that stem cells possess mechanical memory and past matrix stiffness regulates their fate in the future.^[^ ^48^ ^]^ In addition, it is possible that developmentally the d24 hPSC‐LSCs represent more later state LSCs that are proliferating and migrating from limbus toward central cornea and thus prefer stiffer environment. This is also supported by our whole mount data, as d24 hPSC‐LSCs expressed limbal markers CK15 and p40 but also had similar expression of YAP as in the central cornea and high ki67 expression as in the limbal transition zone (Figure 2A,B,H; and Figure S4, Supporting Information). From our standard differentiation protocol on cell culture plastic, Vattulainen et al. showed transient stem cell marker ABCG2 expression already lost in d24 LSCs which in turn have prominent expression of p40 (ΔNp63).^[^ ^30^ ^]^

To test this hypothesis further, we decided to use cells from earlier time point of the hPSC‐LSC differentiation process (Figure 1) and we conducted same analyses for the cells plated at day 5 directly from the embryoid body (EB) suspension culture on different matrices without adherent differentiation and culture on plastic. With this experimental set‐up, we minimized the effect of long‐term culture on stiff plastic and the consequent effect on mechanical memory.

**Figure 5:**
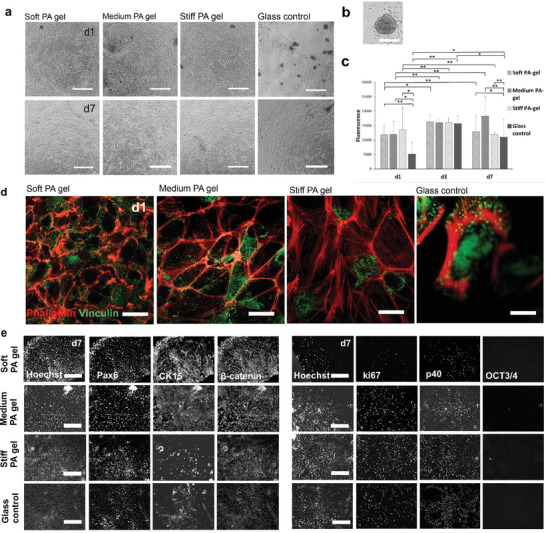
Characterization of embryoid bodies (EB) cultured d5 cells after plating on different stiffness PA‐gel and glass control. a) Phase contrast microscopy images on cell morphology on different stiffness’ at d1 and d7 after plating show good cell adherence on all matrices. b) Phase contrast image of a reference EB not yet plated on adherent culture. c) PrestoBlue viability assay results of EBs hPSC‐LSCs on different stiffness PA‐gels and glass control from d1 to d7 differ significantly from d24 hPSC‐LSC results. Statistical analysis was conducted with nonparametric Kruskal–Wallis H‐test (*n* = 7, ∗∗ = *p* < 0.001, * = *p* < 0.05). d) Immunofluorescence staining of phalloidin (red) and vinculin (green) at d1 after cell seeding show formation of focal adhesions. e) Characterization of relevant LSC marker expression. On left: PAX6, CK15, and β‐catenin cell nuclei visualized with Hoechst. On right: ki67, p40, OCT3/4. Scalebars for images (a) and (e) are 200 µm, for (b) 100 µm and for image (d) 50 µm.

Interestingly, the results are quite opposite than with adherently on plastic cultured later arising d24 hPSC‐LSCs. Morphology images (**Figure** **5**a) show that cells are growing on more densely packed, colony‐like formations on softest gels and cells on two stiffest matrices form less dense cell layer. In addition, PrestoBlue viability assay results indicate significantly lower (*p* < 0.05) viability of cells on glass substrate as compared to cells on all PA gels at d1 (Figure 5c). However, no significant differences in cellular viability were detected between different PA gels at any timepoint. Based on the phalloidin staining (Figure 5d) of the EB cultured cells after plating, cells exhibit more diffuse cytoskeleton organization on soft and medium PA gel, whereas on stiffer substrates (stiff PA gel and glass control), the actin fibers are more pronounced, opposite to d24 LSCs (Figure 4c). As with d24 cells, immunostaining of vinculin showed formation of local adhesions. Overall, the results differ significantly from d24 LSCs, both fresh and defrosted.

For further comparison between different hPSC‐LSC subpopulations, we evaluated protein expression of EB cultured cells after plating and saw that cells are consistently expressing PAX6, CK15, and p40 on all stiffnesses and are negative for OCT3/4 (Figure 5e). Interestingly, CK15 showed most prominent expression on soft PA and medium PA gel, supporting previous findings of Gouveia et al. where matrix softening resulted in higher expression of CK15 in primary human limbal epithelial cells.^[^ ^13^ ^]^ On softest PA gel, ki67 expression was lower than in other matrices, indicating lowest cell proliferation. It has been previously shown that soft matrix supports more quiescent phenotype with low ki67 expression in corneal cells.^[^ ^11^ ^]^ In our study, β‐catenin was showing prominent nuclear localization on glass substrate and on all PA gels, the localization was both cytoplasmic and nuclear, resembling similar staining as in central cornea in whole mount corneas (Figure 2f). As a conclusion, the results show diverse effects of matrix stiffness on hPSC‐LSC marker expression and viability during their early LSC commitment and further differentiation.

### 2.4 Cytoplasmic YAP Correlates with Positive ABCG2 Expression

In corneal cells, mechanosensitive Hippo/YAP pathway has been demonstrated to be a common pathway for both differentiated cells and LSCs to regulate cellular proliferation,^[^ ^49^ ^]^ Interestingly, it has been shown that YAP activation is also contributing to modification of plasticity of mature cells in the cornea.^[^ ^13^ ^]^ In addition, dedifferentiation of mature primary human corneal cells has been shown in 3D Matrigel in vitro in presence of limbal niche cells and after deletion of LSCs in mouse limbus in vivo without damaging the limbal stroma.^[^ ^50^, ^51^ ^]^ These results strongly indicate the important role of soft or native tissue like environment in dedifferentiation.

**Figure 6:**
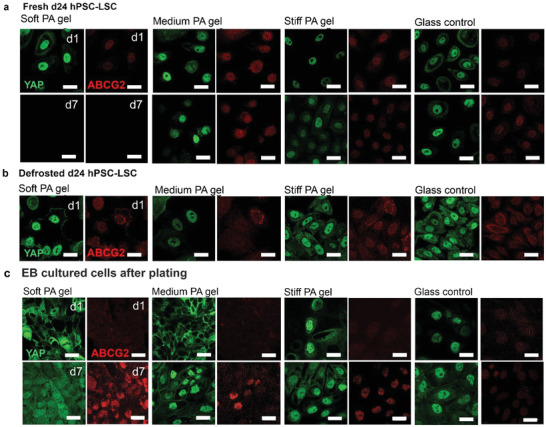
Characterization of Yes‐associated protein (YAP, green) and ATP Binding Cassette Subfamily G Member 2 (ABCG2, red) in hPSC‐LSCs during their differentiation show that soft matrix supports cytoplasmic YAP and promotes ABCG2 expression of EB cultured cells after plating and stiffness alone is not sufficient to induce dedifferentiation of d24 hPSC‐LSCs. Representative image of YAP and ABCG2 for a) fresh d24 hPSC‐LSC, b) defrosted hPSC‐LSC, and c) EB cultured cells after plating on different stiffness PA‐gels and glass control at d1 and d7. Scalebars are 50 µm.

Thus, after the extensive characterization of key corneal markers in native human limbus, we focused on two specific markers, YAP and ABCG2 to see whether d24 hPSC‐LSC exhibit dedifferentiation or to find link between YAP expression and ABCG2, a well‐known stemness marker. As mentioned, we have previously shown that in our hPSC‐LSC differentiation, the expression of ABCG2 begins to rise from early LSC commitment and differentiation and peaks strongly at d11 and is gradually lost at d24.^[^ ^30^ ^]^ In this study, we hypothesize that cells from an early differentiation timepoint (d11) expressing ABCG2 are more likely to represent quiescent LSC phenotype and thus, we could compare YAP expression in LSC populations with known differences in ABCG2 expression in standard culture conditions. We compared YAP and ABCG2 expression in all three LSC populations on varying stiffness matrix via confocal microscopy (**Figure** **6**).

In line with our previous analysis of fresh d24 cells (Figure 4a–d), different matrices did not exhibit differences in YAP or ABCG2 expression in these cells (Figure 6a). On the softest PA‐gel, cells started to detach during culture and at d7, there were no cells left to analyze. Interestingly, even on the softest PA gel at d1, YAP is still exclusively accumulated in the cell nucleus. This localization could support the hypothesis of mechanical memory of the cells. With mesenchymal stem cells, it has been shown that culture conditions that continually induce nuclear translocation of YAP may lead to its constitutive activation, despite of changing mechanical environment.^[^ ^52^ ^]^ On glass control at d7, YAP is localized both in cell nucleus and in cytoplasm and this is also seen in defrosted cells in all matrices at d1 (Figure 6b). One possible reason for this could be that the subcellular localization of YAP is continuous and dynamic process, which appears as rapid nuclear/cytoplasmic shuttling in response to changes in immediate mechanical environment.^[^ ^53^ ^]^ In addition to substrate stiffness, increased cell density also promotes cytoplasmic localization of YAP^[^ ^54^ ^]^ which may contribute in our data to cytoplasmic part of YAP in defrosted d24 hPSC‐LCs and in fresh d24 hPSC‐LSCs on glass at d7 (Figure 6a,b). As expected, the expression of ABCG2 was weak on all matrices and in both fresh and defrosted d24 hPSC‐LSCs (Figure 6a,b). Thus, data suggest that the substrate stiffness alone is not sufficient to induce dedifferentiation and phenotypic changes of d24 hPSC‐LSCs. However, our research setting is missing the other possibly important factors for dedifferentiation, such as secreted factors from other niche cells.

Interestingly, with EB cultured cells after plating, YAP expression on varying stiffness showed a clear stiffness dependent pattern (Figure 6c). On the soft and medium PA gels, the localization of YAP is completely cytoplasmic at d1 and gradually shifts to cytoplasmic and nuclear localization at d7. In medium PA gel, YAP is also strikingly localized in cell junctions at d7, similar to YAP expression in the native human cornea (Figure 2a). On stiff PA gel and on glass control, the localization of YAP is predominantly in the nucleus both in d1 and d7. In our data the soft PA gel, ABCG2 shows notably higher expression compared to stiffer matrices at d7 (Figure 6c). Interestingly, ABCG2 which is essentially a transmembrane efflux pump, is localized also in cell nucleus on all the matrices at that time point. It has been previously reported that in cancer cells, ABCG2 may obtain multiple subcellular locations, including mitochondrial membrane or plasma membrane, depending on the specific function it serves.^[^ ^55^ ^]^ Further studies are required to deepen the understanding of the role of ABCG2 in LSC regulation.

Overall, the results clearly show that soft matrix supports cytoplasmic YAP and maintenance of stem cell phenotype of EB cultured cells after plating, in line with previous reports from primary cells.^[^ ^13^, ^15^ ^]^ As with d24 cells, both fresh and defrosted, variation of substrate stiffness did not induce differential expression of ABCG2 and YAP and hence no evidence for the dedifferentiation was detected based on these markers. In here, different stiffnesses support maintenance of distinct subpopulations, soft one more quiescent type (cytoplasmic YAP) and stiff one more proliferating type (nuclear YAP). Localization and function of YAP in LSCs has been somewhat contradictory. In addition to cytoplasmic retention of YAP and activation of Hippo signaling in soft limbus, it has been also proposed that soft environment promotes nuclear translocation of YAP and maintenance of LSC stemness via downregulated Hippo signaling.^[^ ^44^ ^]^ As a contrary view, it has been suggested that soft substrate promotes primary corneal cell apoptosis.^[^ ^56^ ^]^ Further studies are required to deepen the knowledge in the crosstalk of Hippo signaling with other important signaling pathways in LSCs, although some studies have started to establish this complex route map.^[^ ^12^, ^13^, ^14^ ^]^ For hPSC‐LSCs specifically, further studies should be conducted on d24 cells exclusively differentiated on soft matrix. In addition, further studies should also be carried out to test whether the matrix stiffness in vitro has an effect on the hPSC‐LSC behavior in vivo. As demonstrated in here, differentiation of d24 hPSC‐LSCs on stiff matrix promotes nuclear retention of YAP and highly proliferative phenotype. Potentially, this cell type could have high regenerative capacity in corneal diseases with stiffened corneal matrix and in wound healing conditions. Indeed, our group has previously demonstrated excellent wound healing capacity of d24 hPSC‐LSCs on plastic,^[^ ^9^ ^]^ which supports the statement that the increased wound healing potential of d24 hPSC‐LSCs is dependent of a stiff matrix.

### 2.5 Limbus‐Mimicking Topography Showed Potential in Maintenance of hPSC‐LSC Stemness In Vitro

**Figure 7:**
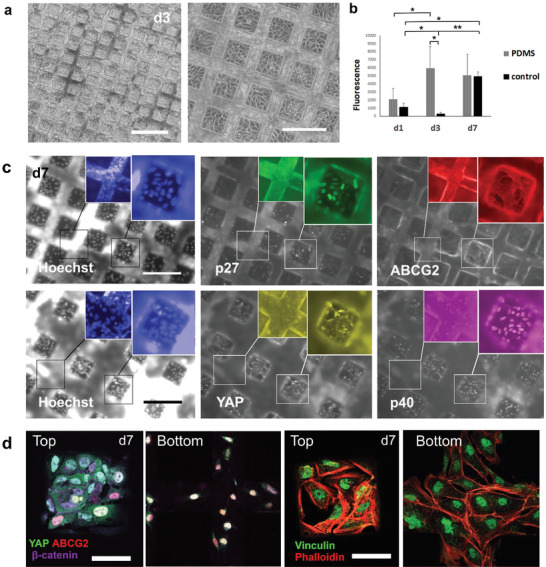
Characterization of EB's cultured on PDMS substrate with a limbus‐mimicking topography show the potential of topography in maintenance of hPSC‐LSC stem cell‐like phenotype in vitro. a) Morphology images of EBs on PDMS substrate at day 3 with 4X and 10X magnification, respectively. b) Prestoblue viability assay results for EB's cultured on PDMS substrate or on glass at day 1 to day 7. Statistical analysis was conducted with nonparametric Kruskal–Wallis H‐test (*n* = 2, ∗∗ = *p* < 0.001, * = *p* < 0.05). c) Protein expression of EBs on PDSM substrates at day 7, imaged with a fluorescence microscope. Hoeshcst (blue), p27 (green), ABCG2 (red), YAP (yellow), and p40 (magenta). d) Confocal microscopy images of relevant protein expression at day 7. Right: YAP (green), ABCG2 (red), β‐catenin (purple) white color indicating coexpression. Left: Vinculin (green), Phalloidin (red). Scalebars are 200 µm a,c) and 50 µm d).

Finally, based on these interesting observations of the influence of matrix stiffness, we studied another important factor in the limbal niche which is the crypt geometry. Limbal area is composed of wave‐like pattern of distinct crypts, for which Grieve et al. reported depth in a range of 15–100 µm.^[^ ^57^ ^]^ Tissue geometry has been shown to have an important role in cell function. As example, Pentinmikko et al. demonstrated that intestinal stem cells, residing in similar crypt‐like niches as LSCs, show reduced regeneration capacity in less‐curved niches, and that their conical shape is an important factor maintaining their functionality.^[^ ^58^ ^]^ Supporting evidence have been published also with primary human LSCs by Prina et al., showing significant differences in cell phenotype within spatial position in gelatin‐based limbus mimicking 3D crypts.^[^ ^59^ ^]^ As we observed in our study that limbus mimicking stiffness supports the stem cell marker expression in EB cultured cells after plating, we selected those for further investigations and cultured them in L‐DOPA+ECM functionalized PDMS substrates patterned with 110 × 110 × 70 µm^3^ cubes, which recapitulate limbal architecture. We studied cellular morphology, viability, and marker expression until d7, results are shown in **Figure** **7**.

Remarkably, EBs attached readily to the PDMS substrate and already at d3, had covered nearly all PDMS substrate area with cell morphology representative of LSC like cells (Figure 7a). In addition, based on PrestoBlue assay, cellular viability on PDMS substrates increases (*p* < 0.05) from d1 to d3 and stabilizes at d7 (Figure 7b). In a flat glass control, viability decreases from d1 to d3 (*p* < 0.05) after which viability increases significantly from d3 to d7 (*p* < 0.001) (Figure 7b). In addition, viability on glass increased significantly from d1 to d7 (*p* < 0.05) which was not seen on PDMS. However, only significant difference in viability between PDMS and glass control was observed at d3 (*p* < 0.001).

In addition to viability and growth, we conducted immunofluorescence staining's for EB cultured cells after plating at d7, to study their marker expression and imaged them with a fluorescence microscope (Figure 7c) and in more detail with a confocal microscope (Figure 7d). On PDMS substrate, cells expressed of ABCG2 and p40 but also notably amount of quiescence marker p27 on squares (Figure 7c). Significant expression of p27 may also explain reduced metabolic activity in PrestoBlue data at d7, indicating a shift towards metabolically inactive, quiescent stage. YAP was localized both in cell nucleus and cytoplasm which was further confirmed with a confocal microscope (Figure 7d). In addition, confocal microscopy confirmed prominent ABCG2 expression as well as β‐catenin localization both in nucleus and in cytoplasm (Figure 7d). Interestingly, ABCG2 and YAP expression was similar as in soft PA gel (Figure 6c) although PDMS is also far from LSCs physiological stiffness (*E* ≈2 MPa).^[^ ^60^ ^]^ Vinculin staining showed also prominent mature focal adhesion formation (Figure 7d). These results suggest that also topography alone may promote hPSC‐LSC stemness. In future studies, it would be interesting to combine both soft matrix and limbal niche topography to evaluate the combinatory effect of these niche factors simultaneously including research questions related to the hPSC‐LSC phenotype in “stiff limbal topography” versus “soft limbal topography” as soft topography would more closely mimic the native tissue.

It must be noted that this study has several limitations. Even with combinatory effect of soft matrix and limbal niche topography presented in this study, only a simplified version of the native limbus is represented. In the native tissue, local structure and composition of the basement membrane are different in the central cornea and limbus which is not taken into account in this study.^[^ ^61^ ^]^ In addition, several important niche elements such as limbal niche cells in proximity of the limbus are neglected in this model.^[^ ^62^ ^]^ In the future, these elements could be implemented to this limbus model. Similar to studies with PA gels, a clear limitation of this study is the short cell culture time on the different matrices. In the future, longer cell culture time would give insight if these mechanobiological platforms are able to support distinct cell phenotype for longer time periods.

## 3 Conclusion

**Figure 8:**
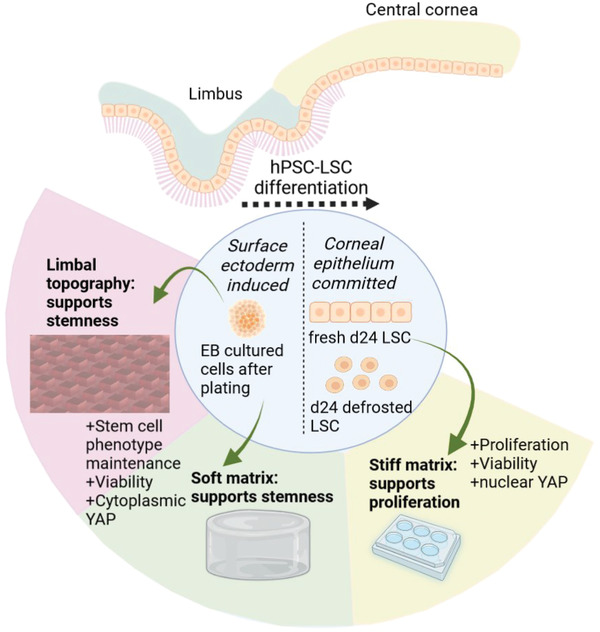
Conclusion of the study. Limbal topography and soft matrix supported stemness of hPSC‐LSCs at early phase of differentiation and stiff matrix supported active proliferation and viability of hPSC‐LSCs at late phase of differentiation. Image created with Biorender.com.

Here, we report an extensive characterization of the effect on matrix stiffness on hPSC‐LSC differentiation and phenotype in vitro with a comparison to native human tissue. We demonstrate differing stiffness‐dependent behavior of hPSC‐LSCs during their differentiation trajectory as summarized in **Figure** **8**.

Our results demonstrate that soft matrix and limbal‐mimicking topography support stemness of hPSC‐LSCs at early phase of differentiation in vitro. On the contrary, hPSC‐LSCs at late stage of differentiation prefer stiff matrix which supported their proliferation and viability in vitro. Soft matrix could not induce dedifferentiation of d24 LSCs, indicating a strong preference on the mechanical properties of the culture matrix.

Overall, our results are suggesting that hPSC‐LSCs create mechanical memory during their differentiation, and this can be driven by adherent culture condition or by developmental state of the differentiating cells which an important property of the cells in vivo. Our findings highlight the importance of the cellular mechanical memory as a key factor for cell and cellular microenvironment interactions, which has been largely neglected before and as a crucial design element of biomaterials for cell culture and transplantations as well as in vitro modeling of limbal niche.

## 4 Experimental Section

### hPSC‐LSC Differentiation

All studies were conducted with a supportive statement (Skottman/R05116) from the Ethics committee of the Pirkanmaa Hospital district, Tampere, Finland. hPSC‐LSCs were differentiated from human embryonic stem cell line (Regea08/017) as previously described by Hongisto et al. and utilized in the study in three different time points during differentiation process which were d5, d24 and d24 after cryostorage (Figure 1).^[^ ^10^ ^]^ Briefly, blebbistatin (Merck) (10 µL, 5 µm) was added to single cell suspension of pluripotent stem cells to induce embryoid body (EB) formation, following four additional days of induction toward surface ectoderm in defined XF‐Ko‐SR medium (Thermo Fischer) (10 mL). In fifth day of induction, EBs were either utilized directly in the study by plating on different matrices or transferred onto plates coated with 0.5 µg cm^−2^ recombinant laminin‐521 (LN‐521, Biolamina, Sweden) (0.1 mg mL^−1^) and 5 µg cm^−2^ human placental collagen Type IV (Col IV, Merck) (1 mg mL^−1^) for further adherent differentiation in commercial Cnt‐30 medium (CELLnTEC Advanced Cell Systems AG, Bern, Switzerland). Adherent differentiation was carried on for 19 more days with medium three times a week and after 24 days of differentiation, cells were detached with TrypLE Select Enzyme (Thermo Fischer) and further seeded on materials (fresh d24 hPSC‐LSC population) or cryopreserved in PSC Cryopreservation medium (Thermo Fisher). Third cell population was thawed directly from cryostorage to the PA gels (defrosted d24 hPSC‐LSC population). All experiments are conducted with Regea 08/017 cell line and detailed information of experimental sample number (n) is described for each analysis method separately.

### Polyacrylamide Gel Synthesis

Polyacrylamide gels (PA gels) with three different stiffnesses were synthesized with previously published protocol by Tse and Engler with slight modifications.^[^ ^17^ ^]^ Briefly, 15 mm coverslips (bottom glass) and 13 mm coverslips (upper glass) were cleaned with 2% Hellmanex cleaning solution (Merck) for 1 h in +37 °C and rinsed with EtaX Aa (Altia Finland) and Milli‐Q water and let air‐dry in a fume hood. For adhesion of PA gels to bottom glasses, coverslips were treated with a solution of 3‐(Trimethoxysilyl)propyl methacrylate (Bind‐Silane, Merck) (3 µL), glacial acetic acid (50 µL), and Etax Aa (950 µL, ≥99.5%) for 3 min in RT. After treatment, coverslips were rinsed twice in Etax Aa and let air‐dry in a fume hood. Bottom glasses were stored for maximum 3 months in a desiccator before use. Upper glasses were treated with poly(*L*‐lysine)‐graft‐poly(ethylene glycol) copolymer (PLL‐*g*‐PEG, SuSoS AG, Dübendoft, Germany) (30 µL, 0,1 mg mL^−1^) in PBS for 45 min in RT to prevent adhesion of PA gel surface. Coverslips were rinsed twice with milliQ‐H20 and stored in PBS for maximum 7 days in +4 °C prior use. PA gels with different stiffnesses were prepared between the coverslips according to **Table** **1**.

**Table 1:** Preparation of PA gels with varying stiffness

	Soft PA gel [µL]	Medium PA gel [µL]	Stiff PA gel [µL]
10X PBS	500	500	500
40% Acrylamide	375	1250	1250
2% Bis	250	250	750
H_2_O	3875	3000	2500
Tot. vol	5000	5000	5000

Monomer solutions were then degassed for ≈3 min to get rid of free oxygen which interferes with polymerization. After degassing, polymerization was initiated with addition of tetramethylethylenediamine (TEMED, Merck) (10 µL) and 10% ammonium persulfate (APS, Merck) (50 µL). A 13 µL drop of the solution was pipetted in the center of a bottom glass to result in ≈100 µm thick gel and an upper glass was inserted on top. PA gels were let to polymerize for 30 min in RT and afterward immersed in PBS. Gels were soaked in PBS in +4 °C at least overnight and washed in several steps during the surface functionalization to release any unreacted acrylamide from the gels due its neurotoxicity.^[^ ^63^ ^]^ Gels were stored in +4 °C for maximum 7 days. Upper glass as gently removed with a scalpel and PA gels were sterilized with a germicidal lamp for 30 min prior coating and cell seeding.

### Preparation of PDMS Substrate with Limbus Mimicking Topography

The molds were fabricated from SU‐8 photoresistive epoxy on silicon wafers using rapid prototyping.^[^ ^64^ ^]^ Two molds were designed using AutoCad (AutoDesk), both designs contained 110 × 110 µm^2^ covering 100 mm wafer, in the first mold the squares were spaced 110 µm apart, and in the second the squares were spaced 55 µm apart. These designs were used to fabricate two 125 × 125 mm^2^ chrome on glass masks using µpg501 direct writing system (Heidelberg Instruments Mikrotechnik GmbH). 70 µm high SU‐8 features were fabricated from SU‐8 3050 (Kayaku Advanced Materials Inc) on top of 100 mm silicon wafers (Universitywafer Inc) using standard UV photolithography. PDMS substrates were prepared from ready to use Sylgard 184 Clip‐bags (Merck). Monomer and curing agent were mixed and degassed with a vacuum pump. Degassed solution was poured on a mold and let cure for 10 h in +60 °C before peeling the PDMS off and cutting into 15 × 15 mm^2^ substrates with a scalpel.

### Matrix Coating

To coat PA gels and PDMS substrates with ECM proteins to promote cell adhesion, a polydopamine coating was first prepared to functionalize gel surface according to the protocol by Wouters et al.^[^ ^18^ ^]^ Briefly, catecholamine L‐DOPA (Merck) (16 mg, 2 mg mL^–1^) was dissolved in 0.1 m Tris‐buffer pH 10 (WVR) (8 mL) for 30 min in the dark. L‐DOPA solution was sterilized with a 0.1 µm Pall Acrodisc syringe filter (Merck) and gels were incubated with L‐DOPA solution (250 µL) in RT for 30 min in the dark. After that, gels were washed twice with PBS to remove residual L‐DOPA and coated with 5 µg cm^–2^ Col IV and 0.5 µg cm^–2^ LN‐521 for 3 h in +37 °C prior cell seeding. For all material characterization (AFM and rheological analysis), one gel sample is considered *n* = 1.

### AFM Measurements

The Young's moduli of the varying stiffness PA gels were determined by indentation using a MultiMode 8 atomic force microscope equipped with a NanoScope V controller and a closed‐loop PicoForce scanner (Bruker, Santa Barbara, CA). The gels with or without protein coating, were indented with the tip of a MLCT probe (Bruker; probe D was used, nominal tip radius 20 nm) at a speed of 2 µm s^−1^, applying a maximum force of 5 nN. Previously, the deflection sensitivity of the system was determined by measuring the deflection of the probe against a mica substrate. The spring constant of the probe (0.066 N m^−1^) was obtained with the thermal tune method.^[^ ^65^ ^]^ The experiments were carried out in PBS at room temperature. Between 30 and 60 indentation curves were recorded for each PA gel which were collected from different spots (between 3 and 6) on the same or two different samples. The Young's moduli were obtained by fitting the approach indentation curves with the Sneddon model,^[^ ^66^ ^]^ (linearized equation) using the software NanoScope Analysis 1.5 (Bruker). The values for the half‐angle of the tip and the Poisson's ratio used in the fits were 18° and 0.3°, respectively.

### Rheological Measurements

Rheological characterization of the variable stiffness PA gels was performed with DHR‐II hybrid rheometer (TA Instruments) with a 12 mm parallel plate geometry at +21 °C. For rheological studies, PA gel samples were prepared in a cut 10 mL syringes. Oscillatory frequency sweep was conducted to characterize mechanical properties of the bulk gels. Oscillatory frequency sweep was conducted within linear viscoelastic region (LVE) with a constant strain of 1% and with frequency ranging from 0.1 to 10 Hz. Storage modulus (*G’*) was plotted against this frequency range. Damping factor tan(*δ*) was determined as a ratio between loss (*G’’*) and storage modulus. Frequency sweep was conducted from triplicate samples (*n* = 3).

### Cell Seeding and Culture on Materials

EB's were plated on varying stiffness PA‐gels and PDMS substrates by adding ≈15 same sized EBs per cm^2^ and cultured in Cnt‐30 medium for 7 days. Same cell seeding density, 50 000 cells cm^−2,^ was utilized with fresh and cryostoraged d24 hPSC‐LSCs and both populations were cultured on PA‐gels for 7 days in Cnt‐30 medium with a medium change every other day. In vitro analyses were conducted in time points d1, d3, and d7. For in vitro analysis, one gel sample with cells are considered *n* = 1.

### Cell Viability and Growth

The viability and growth of hPSC‐LSCs on the varying stiffness PA gels and PDMS substrates were evaluated in d1, d3, and d7 based on cell morphology with a phase contrast microscope Nikon Eclipse TE2000‐S (Nikon Instruments, Netherlands). Additionally, the viability was quantitatively evaluated based on cell metabolism with PrestoBlue viability assay (Merck) in time points d1, d3, and d7 according to manufacturer's instructions. Briefly, PrestoBlue reagent was diluted with 1:10 v/v in cell culture medium (40 µL) and was added to the samples (400 µL). Samples were incubated for 30 min in +37 °C and medium (100 µL) was collected from one sample in triplicates (considered as three technical replicates). Fluorescence was measured with Viktor 1420 Multilabel Counter (Wallac, Turku, Finland) at 544 nm excitation and 590 emission wavelengths. Blank samples were prepared to subtract background fluorescence from the results. All experiments were conducted for fresh d24 hPSC‐LSCs and EBs with seven samples per each varying stiffness matrix (*n* = 7). For defrosted d24 hPSC‐LSCs, experiments were conducted with three samples (*n* = 3).

### Donor Cornea Processing

Human donor corneas, classified unsuitable for transplantation, were received from Regea Tissue Bank, Tampere University, Finland in CorneaMax medium (EuroBio Scientific, Les, Ulis, France) or CARRY‐C (Alchimia, Ponte San Nicoló) and processed under supportive statement (Skottman/R11134) from Ethics committee of the Pirkanmaa Hospital district, Tampere, Finland. Corneas were obtained from 6 donors (donor age varied from 53 to 76 years) and corneas were used 1–30 days after detachment. Limbal crypts were identified as a striped pattern in the corneoscleral border under a stereo microscope and ≈4 × 7 mm^3^ pieces were cut from the corneas. Tissue pieces were washed once with PBS and fixed with 4% PFA (1 mL) for 30 min in RT.

### Immunohistochemistry

To evaluate protein expression of hPSC‐LSCs on different matrices, cells were washed once with PBS and fixed with 4% PFA (500 µL) for 20 minin RT, following two PBS washes and storage in +4 °C before indirect immunofluorescence staining which was performed as previously published by Mikhailova et al.^[^ ^67^ ^]^ List of primary antibodies and corresponding Alexa Fluor 488, 568, or 647 conjugated secondary antibodies can be found from the Supporting Information (Table S1). Samples were mounted with Vectashield Antifade Mounting Medium (Vector Laboratories) between two coverslips and sealed with nail polish. Imaging of the samples was performed with Zeiss LSM 800 confocal laser scanning microscope (Zeiss, Jena, Germany) with a 63X/1.4 oil immersion objective or Olympus IX51 fluorescence microscope (Olympus Corporation, Japan).

Whole mount immunofluorescence staining of human donor cornea pieces was performed to study corresponding protein expression in vivo with slight modifications to a protocol presented by Renner et al.^[^ ^68^ ^]^ All steps in whole mount staining were performed in RT. After fixing, human donor corneas were washed once with DPBS, following blocking and permeabilization in PBS with 0.5% Triton X‐100 (Sigma‐Aldrich) (2.5 mL, 4%), 3% Bovine serum albumin (BSA, Sigma‐Aldrich) (6 mL, 10%), and 0.02% NaN_3_ (0.2 mL, 2%) (blocking buffer) overnight. Next day, samples were incubated with primary antibodies (Table S1, Supporting Information) in blocking buffer for 7 days. Antibody solution was replaced every 2 days. Samples were washed with blocking buffer for 4 days with a daily change of the solution. After washing, secondary antibodies were applied in blocking buffer for 7 days. Antibody solution was replaced every 2 days. Next, samples were washed using PBS with 1:1000 Hoechst (Invitrogen) for 3 days to visualize cell nuclei. Solution was changed daily. In the last day of the staining protocol, samples were dehydrated in a dilution series of methanol (Merck). Samples were immersed 20%, 35%, 50%, 75%, and 100% methanol in milliQ‐H20 for 20 min in each dilution. To make samples transparent, samples were first transferred to 10 mL glass containers and then immersed in 50:50 methanol: 2:1 benzyl benzoate:benzyl alcohol (BABB) solution for 30 min. Finally, samples were immersed in 100% BABB and stored in dark in RT before imaging. Imaging of the samples was performed with Zeiss LSM 800 confocal laser scanning microscope (Zeiss, Jena, Germany) with a 25X/0.8 oil immersion objective. Image processing was carried out with Zeiss Blue Software and Corel PhotoPaint Graphics Suite. Confocal images of the whole mount immunofluorescence staining's were deconvoluted utilizing Hyugens Essential deconvolution Wizard software (Scientific Volume Imaging).

### Statistical Analysis

Statistical analysis for PrestoBlue viability assay results (Figures 4b,f, 5b, and 7b, data presented as mean with standard deviation) was conducted with IBM SPSS Statistics Software with nonparametric Kruskal–Wallis H‐test. Sample size for d24 hPSC‐LSC viability was *n* = 7 (Figure 4b), for defrosted hPSC‐LSC viability *n* = 3 (Figure 4f) and for embryoid bodies (EB) cultured d5 cells after plating *n* = 7. *p* < 0.05 was considered statistically significant.

## Conflict of Interest

The authors declare no conflict of interest.

## Supporting information

Supporting Information

## Data Availability

The data that support the findings of this study are available from the corresponding author upon reasonable request.
