# Supplementary material for: Toward Corneal Limbus In Vitro Model: Regulation of hPSC‐LSC Phenotype by Matrix Stiffness and Topography During Cell Differentiation Process
Source: Adv Healthc Mater. 2023 Jul 21;12(29):2301396. doi: 10.1002/adhm.202301396 (PMC11468526; doi:10.1002/adhm.202301396)
Supplement: Supplementary file 1 — Supporting Information [file ADHM-12-2301396-s001.pdf]

ADVANCED HEALTHCARE MATERIALS

Supporting Information

for *Adv. Healthcare Mater.*, DOI 10.1002/adhm.202301396

Toward Corneal Limbus In Vitro Model: Regulation of hPSC-LSC Phenotype by Matrix Stiffness and Topography During Cell Differentiation Process

*Maija Kauppila, Anni Mörö, Juan José Valle-Delgado, Teemu Ihalainen, Lassi Sukki, Paula Puistola, Pasi Kallio, Tanja Ilmarinen, Monika Österberg and Heli Skottman**

Supporting Information

Towards corneal limbus in vitro model: regulation of hPSC-LSCs phenotype by matrix stiffness and topography during cell differentiation process.

*Maija Kauppila, Anni Mörö, Juan José Valle-Delgado, Teemu Ihalainen, Lassi Sukki, Paula Puistola, Pasi Kallio, Tanja Ilmarinen, Monika Österberg, Heli Skottman**

Table 1. Antibodies and their dilutions

Antibody	Manufacturer	Host	Dilution
ABCG2	Millipore (MAB4155)	Mouse	1:200 (cell analysis)
	Abcam (ab229193)	Rabbit	1:200 (whole mount)
β-catenin	R&D (AF1329)	Goat	1:200
CK15	Thermo Fischer (MS-1068-P1)	Mouse	1:200
ki67	Millipore (AB9260)	Rabbit	1:200
OCT3/4	R&D (AF1759)	Goat	1:200
PAX6	Sigma (HPA030775)	Rabbit	1:200
p27	Abcam (ab321034)	Rabbit	1:200
p40	Biocare Medical (ACI3066C)	Mouse	1:100
Vinculin	Sigma (V4139-200UL)	Rabbit	1:100
YAP	Thermo Scientific (PA1-46189)	Rabbit	1:500
Alexa Fluor 488 a-rabbit	Molecular Probes (A21206)	Donkey	1:800 (cell analysis) 1:400 (whole mount)
Alexa Fluor 568 a-mouse	Molecular Probes (A10037)	Donkey	1:800 (cell analysis) 1:400 (whole mount)
Alexa Fluor 647 a-goat	Abcam (AB150131)	Donkey	1:800 (cell analysis) 1:400 (whole mount)
Phalloidin–Tetramethylrhodamine B isothiocyanate	Sigma (P1951)		1:100

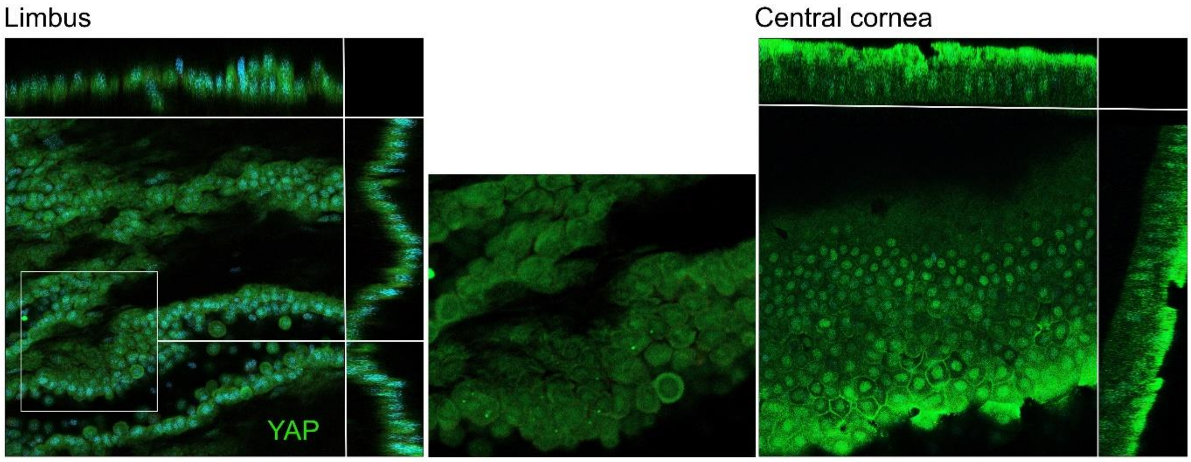

Figure S1. A Z-stack image of YAP expression in human limbus and central cornea. Cell nuclei are counterstained with Hoechst (blue).

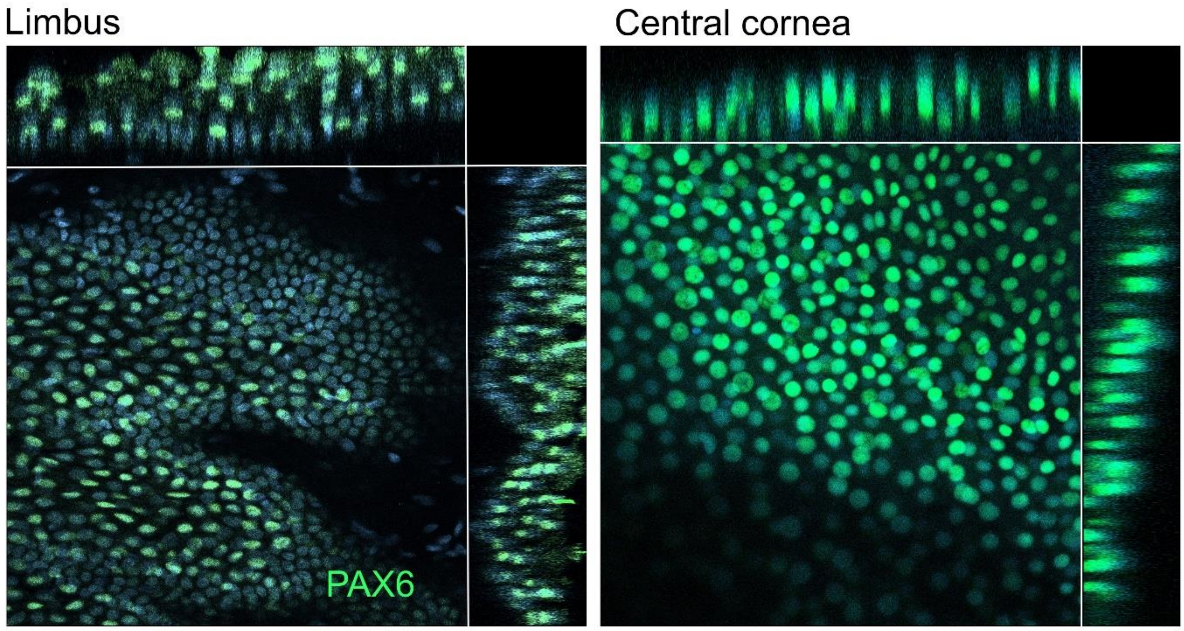

Figure S2. A Z-stack image of Pax6 expression in human limbus and central cornea. Cell nuclei are counterstained with Hoechst (blue).

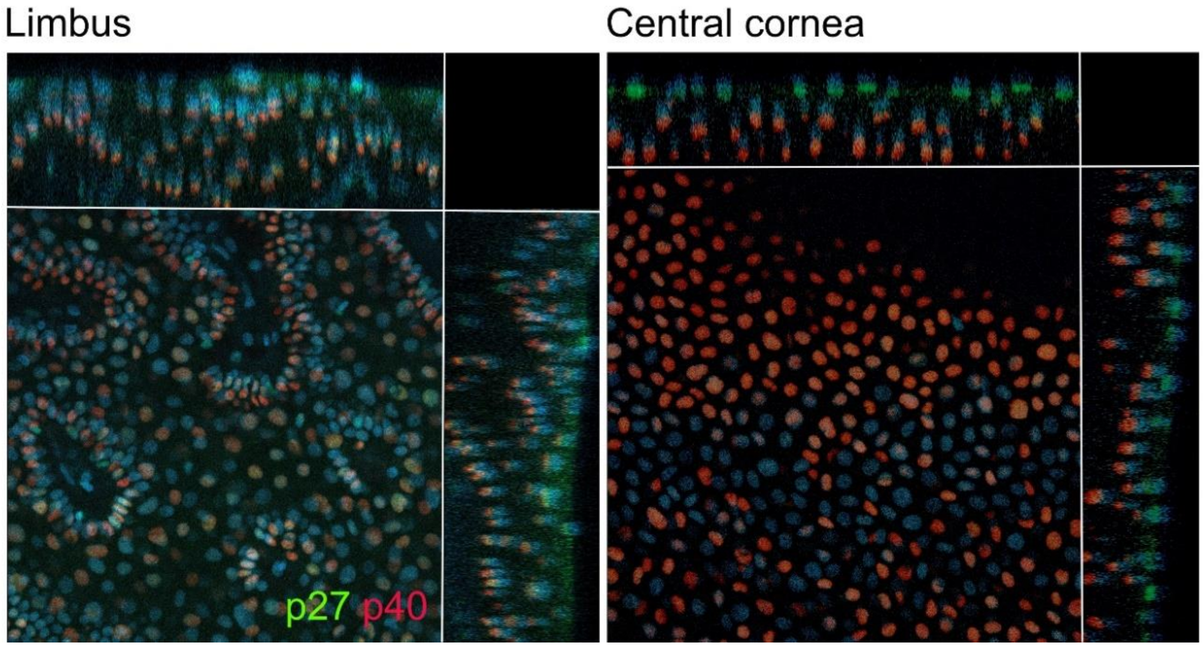

Figure S3 A Z-stack image of p27 and p40 expression in human limbus and central cornea. Cell nuclei are counterstained with Hoechst (blue).

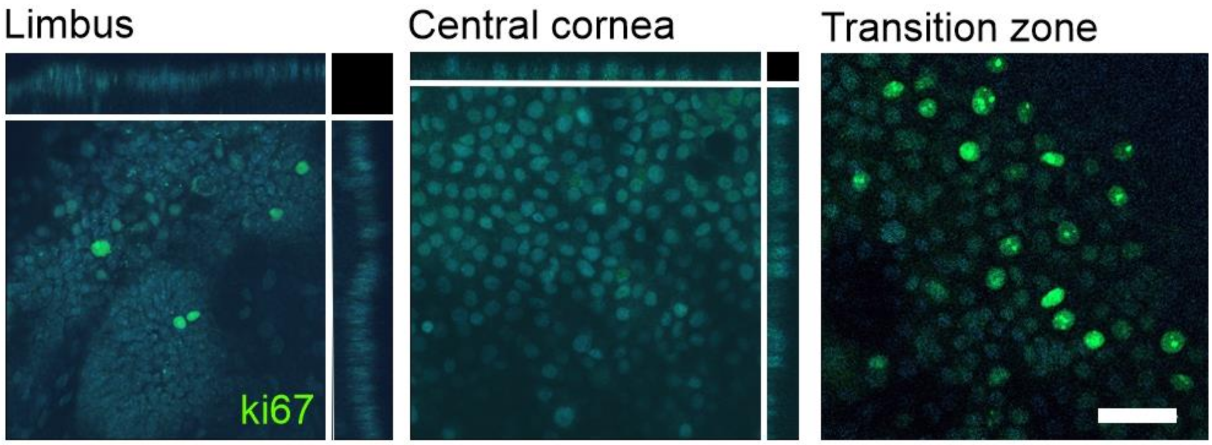

Figure S4. A Z-stack image of ki67expression in human limbus, central cornea and in transition zone between limbus and central cornea, respectively. Cell nuclei are counterstained with Hoechst (blue). Scale bar is 50 µm.

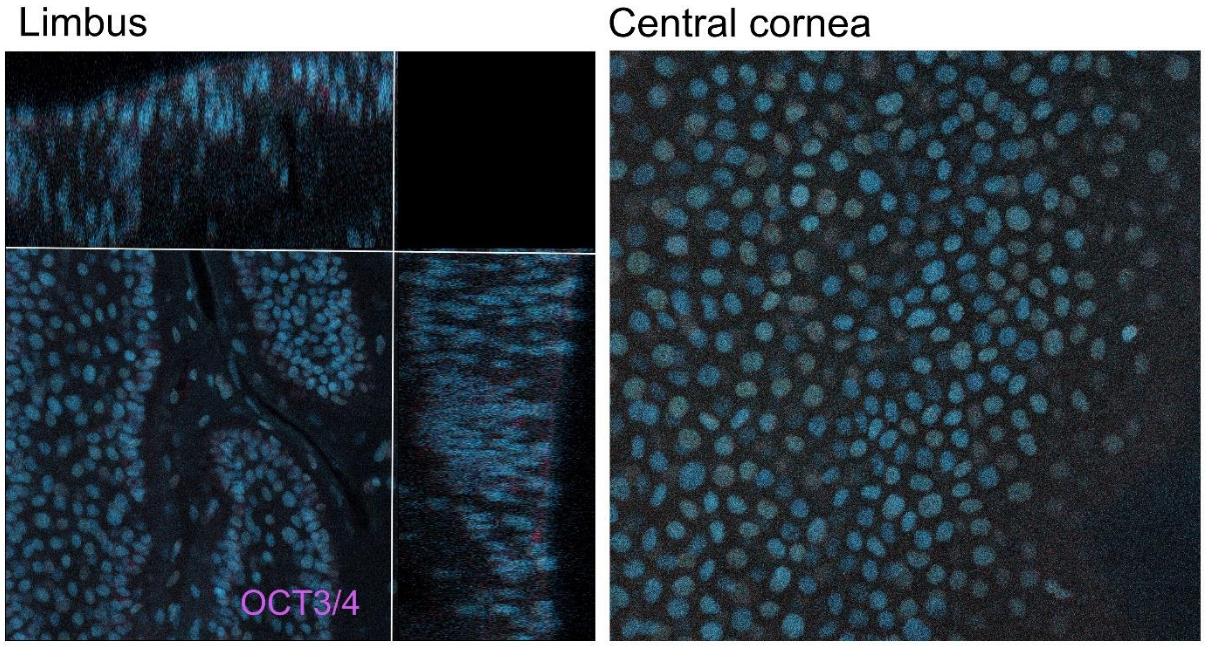

Figure S5. A Z-stack image of OCT3/4 expression in human limbus and central cornea (negative throughout). Cell nuclei are counterstained with Hoechst (blue).

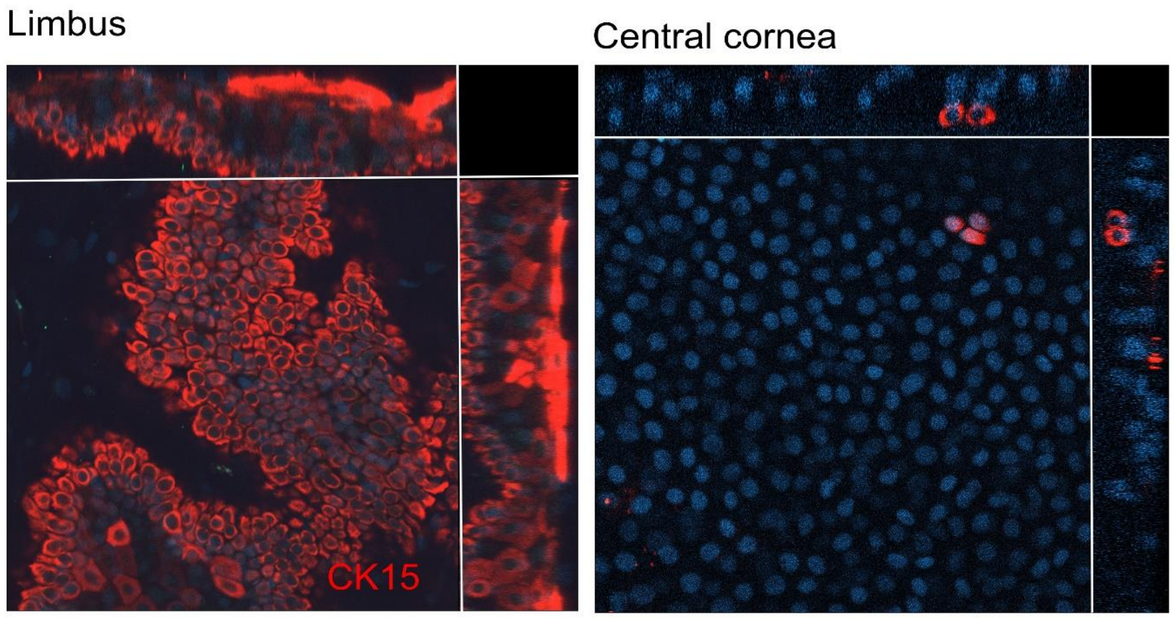

Figure S6. A Z-stack image of CK15 expression in human limbus and central cornea. Cell nuclei are counterstained with Hoechst (blue).

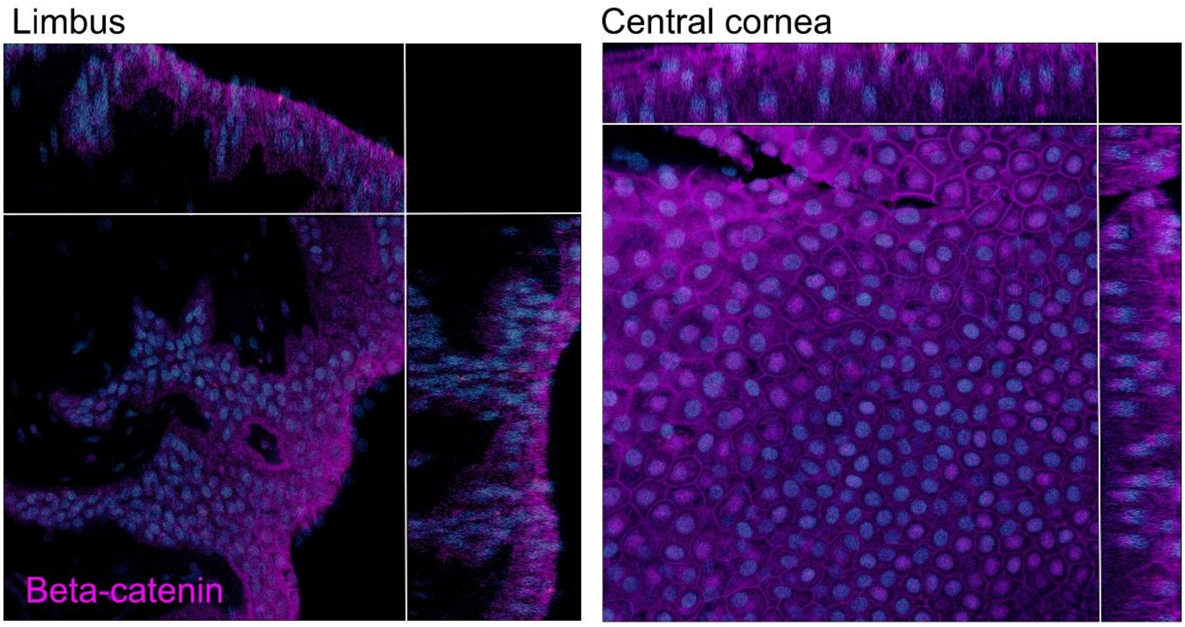

Figure S7. A z-stack image of β-catenin expression in human limbus and central cornea. Cell nuclei are counterstained with Hoechst (blue).

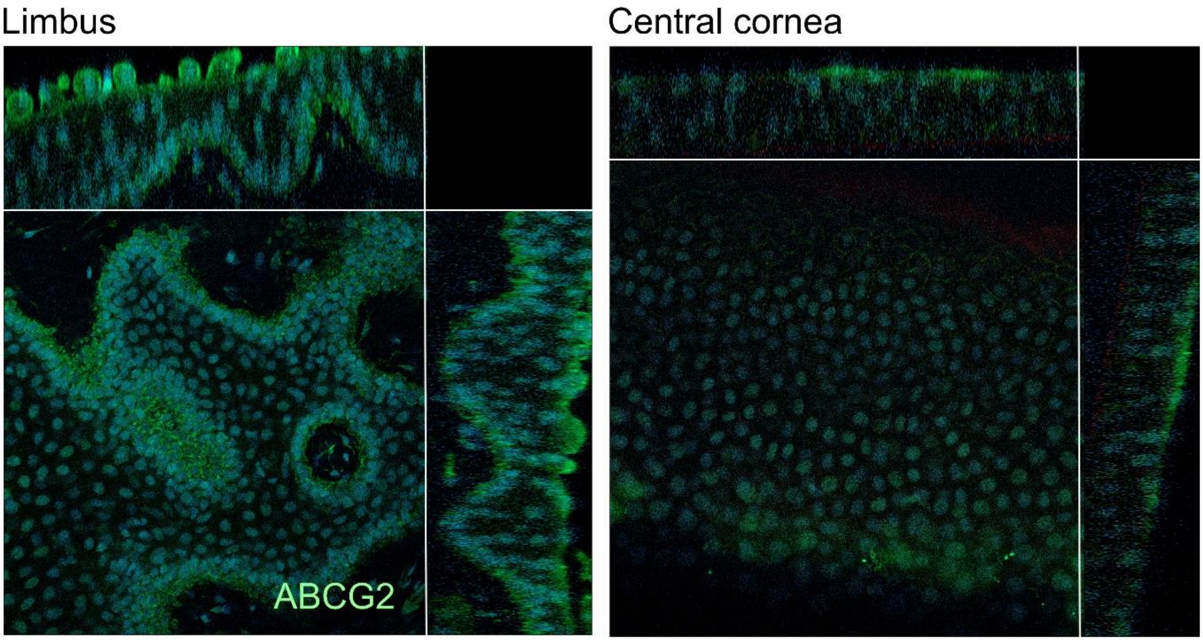

Figure S8. A Z-stack image of ABCG2 expression in human limbus and central cornea. Cell nuclei are counterstained with Hoechst (blue)
